# Predictors of family functionality amongst human immunodeficiency virus-serodiscordant couples in two major hospitals in Kumasi, Ghana

**DOI:** 10.4102/phcfm.v12i1.2355

**Published:** 2020-06-09

**Authors:** Nana K. Ayisi-Boateng, Anthony Enimil, Aliyu Mohammed, Akye Essuman, Henry Lawson, Douglas Opoku Aninng, David Agyemang-Yeboah, Kathryn Spangenberg

**Affiliations:** 1 Department of Medicine, School of Medicine and Dentistry, Kwame Nkrumah University of Science and Technology, Kumasi, Ghana; 2 University Hospital, Kwame Nkrumah University of Science and Technology, Kumasi, Ghana; 3 Department of Child Health, School of Medicine and Dentistry, Komfo Anokye Teaching Hospital, Kumasi, Ghana; 4 Department of Epidemiology and Biostatics, School of Public Health, Kwame Nkrumah University of Science and Technology, Kumasi, Ghana; 5 Family Medicine Unit, Department of Community Health, University of Ghana Medical School, Accra, Ghana; 6 Family Medicine Directorate, Komfo Anokye Teaching Hospital, Kumasi, Ghana

**Keywords:** APGAR, family functionality, predictors, serodiscordance, HIV

## Abstract

**Background:**

Family instability and partner conflicts are reportedly common in serodiscordant relationships. To date, the family adaptability, partnership, growth, affection and resolve (Family APGAR), a standardised tool for assessing family function, has not been used in any published literature involving this peculiar group.

**Aim:**

The aim of this study was to determine the predictors of family functionality and its association with human immunodeficiency virus (HIV) serodiscordance.

**Setting:**

The study was undertaken at the Kwame Nkrumah University of Science and Technology Hospital and Komfo Anokye Teaching Hospital in Kumasi, Ghana.

**Method:**

This was a cross-sectional study. A systematic sampling method was used to select HIV-positive clients whose partners were seropositive (concordant) or seronegative (discordant). A standardised format was used to extract relevant data. All data were analysed using STATA^®^ (version 14). Results were reported as odds ratios with 95% confidence intervals for study and outcome variables.

**Results:**

The study recruited 374 respondents, of which 52% (195) were in HIV-discordant relationships. Approximately 68% (254) of the respondents rated their families as functional, 15% (57) rated as moderately dysfunctional and 17% (63) rated as severely dysfunctional. A statistically significant relationship was found between family functionality and gender, as well as between family functionality and HIV status disclosure to the partner. No association was found between the Family APGAR and HIV serodiscordance.

**Conclusion:**

Amongst HIV couples, the strongest predictors of family functionality are gender and status disclosure. Healthcare providers should invest efforts into addressing gender-based challenges, utilise the Family APGAR and support disclosure of HIV status, especially amongst discordant couples.

## Introduction

Any sexual relationship in which one partner is human immunodeficiency virus (HIV)-positive and the other is HIV-negative is termed serodiscordant, mixed status, serodivergent or, simply, discordant.^1^ The World Health Organization (WHO) estimates that of HIV-positive patients who are in a sexual relationship, 50% have a ‘mixed status’. The prevalence of HIV discordance amongst couples in sub-Saharan Africa is between 3% and 20% in the general population.^1^ Generally, in most studies involving discordant couples, there is a 50:50 male-to-female proportion.^2^

Socio-economic factors such as gender, race or ethnicity and other social determinants of health have been identified as crucial in the fight against HIV and acquired immunodeficiency syndrome (HIV and AIDS).^3^ Apart from the economic burden it presents, HIV infection threatens the stability of families, especially amongst serodiscordant couples. Marriage dissolution, suspicion of infidelity and partner separation frequently occur amongst people living with HIV, and HIV-infected women in discordant marriages are more likely to face divorce.^4^ A study conducted in Malawi showed that marriage dissolution was highest in discordant couples in which the woman was HIV-positive.^5^

Human immunodeficiency virus infection, like other acute or chronic illnesses, can be a source of stress for individuals and their family.^6^ While a patient’s health can affect the family or caregiver, family challenges can also affect the patient’s disease outcome.^6^ There is clinical and empirical evidence to support the fact that family problems can be a significant source of stress for people living with HIV, second only to stress because of the disease itself.^7^ Family support is critical in the care of patients because it provides the needed psychological, emotional and financial support both at home and in the hospital.^7,8^ The importance of a functioning family for the positive clinical outcome of an HIV-positive patient, therefore, cannot be overemphasised.

The Family APGAR questionnaire was designed by Smilkstein in 1978 and has been revised to test five areas of family function, which include adaptability, partnership, growth, affection and resolve.^9^ A total score is assigned to each respondent, and a score of 7–10 is classified as highly functional family, a score of 4–6 is classified as moderately dysfunctional family and a score of 0–3 is classified as severely dysfunctional family. Its validity and reliability have been tested and it is now widely accepted as a useful instrument for clinical practice and research.^9^

The aim of this study was therefore to determine socio-demographic predictors of family functionality (using the Family APGAR), compare family functionality as assessed by patients in discordant relationships with those in concordant relationships and establish whether there are any associations.

## Methods

This was a hospital-based cross-sectional study conducted at the Infectious Disease Unit (IDU) of the Kwame Nkrumah University of Science and Technology (KNUST) Hospital and the Chest Clinic of Komfo Anokye Teaching Hospital (KATH) in Kumasi in the Ashanti Region of Ghana.

Kwame Nkrumah University of Science and Technology Hospital is a quasi-government institution located on the campus of KNUST. It is a 100-bed hospital and is considered a district hospital, and provides both general and specialist services. The IDU of the hospital was set up in 2010 and has since then been attending to patients diagnosed with HIV and/or AIDS, hepatitis B and C, tuberculosis and other infectious diseases. The unit has approximately 1200 registered clients suffering from HIV and AIDS. On the two clinic days, on average, 30 patients are seen per day (approximately 60 patients per week). Annually, a total of 2880 clients are seen at the IDU, with an average of 240 patients a month. Patients who report on clinic days are taken through drug adherence counselling, counselling on safe sex practices, partner counselling and testing, and family planning methods. There are also HIV-positive volunteers who offer peer social support to clients at the unit.

Komfo Anokye Teaching Hospital is the only teaching hospital within the Ashanti Region. It is the second largest teaching hospital in Ghana after Korle Bu Teaching Hospital in Accra. The hospital serves as a major referral point for health facilities in Ashanti and other regions of the country. It was established in 1955 and became a teaching hospital in 1975. Komfo Anokye Teaching Hospital has a capacity of approximately 1200 beds. It is located in Bantama, a densely populated area, near Kumasi City Centre. The Chest Clinic, the biggest Antiretroviral therapy centre in Kumasi, was set up in 2003. It provides services for patients suffering from HIV and AIDS, as well as for those with tuberculosis. Since the inception, a total of 14 571 patients have been registered at this facility. On average, about 1000 patients are seen in a month and February 2017 alone recorded 1484 patients. On average, 60 patients are seen on each clinic day.

## Study population and sample selection

The study participants were defined as HIV-positive clients, who were 18 years old and above, married or cohabiting, knew the HIV status of their partners and were registered clients at the two study sites.

### Inclusion criteria

This study used the following inclusion criteria:

1. Participants must be HIV-positive (male and female candidates) and 18 years old and above.
2. Participants should be married or cohabiting.
3. Human immunodeficiency virus status of the participant’s partner must be known.
4. Participant must sign an informed consent form.

### Exclusion criteria

The following exclusion criteria were used in this study:

1. Patients who were too ill to participate.
2. Human immunodeficiency virus-positive patients on hospital admission.

### Sample size calculation

The sample size was calculated using a baseline study conducted in Cape Coast, Ghana, which showed a percentage of 58.2 of HIV-positive patients being either concordant or discordant.^10^ It was based on a confidence interval (CI) of 95% and a 5% allowable margin of error. The following formula was used for the calculation:^11^

$$\text{Sample size}=\frac{{\text{Z}}^{2}(\text{P})(1-\text{P})}{{\text{E}}^{2}}$$ [Eqn 1]

where *Z* = the number relating to the degree of confidence. The standard score for the 95% CI is 1.96.

*P* = an estimate of the proportion of HIV discordance and concordance in Ghanaian couples, which is approximately 58.2%.^10^

*E* = allowable margin of error that is 5%:

$$\begin{array}{ll} \text{Then the sample size} & =\frac{{\left(1.96\right)}^{2}(0.582)(1-0.582)}{{\left(0.05\right)}^{2}}\\ \; & =\frac{3.842\times 0.582\times 0.418}{0.0025}\\ \; & =\frac{0.935}{0.0025}\\ \; & \approx 374 \end{array}$$ [Eqn 2]

On average, 30 HIV-positive patients are seen at the IDU of the KNUST Hospital on a clinic day, while 60 patients are seen each day at the Chest Clinic of KATH. It is estimated that out of a population of HIV-positive patients at an ART clinic, 58.2% are in relationships who may be described as either concordant or discordant.^10^ Hence, before the study was conducted, it was projected that at the KNUST Hospital, approximately 17 participants per day (58.2% of 30) potentially be qualified to be recruited and 35 participants (58.2% of 60) at KATH. Hence, for both study sites, the *n*th folder that was selected for patients reporting to the clinic was 2 (30/17 for KNUST and 60/35 for KATH).

Participants were selected after careful screening of all patients who reported to the ART centres on their scheduled clinic days, to see a clinician or to refill their medications. A systematic sampling method was employed in recruiting 374 study participants. When the patients reported to the two sites on their clinic days, their folders were retrieved as is routinely performed and arranged in a pile; the folder of a patient who reported first on a clinic day was placed on top and followed in order of time of reporting. To determine which folder to pick first and at what interval (at either study site), balloting was used; folded pieces of paper marked individually with numbers ‘1’ and ‘2’ were placed in a basket and one of them was picked at random. The number that was randomly selected was number ‘2’.

Hence, the first folder was not selected, but the second one was selected and every other folder till the last folder. The patient of a selected folder was carefully screened to ensure that he or she fitted into the inclusion criteria. Folders of patients who did not fit the inclusion criteria were excluded from the study and the next folder based on the pre-determined interval was chosen.

The consent process was carried out on an individual basis. Twi or English, the two dominant languages spoken in the geographical area, was used.

A participant data capture sheet was designed to capture all participants’ information, such as age, sex, marital status and ART centre. The Family APGAR questionnaire, incorporated into a structured questionnaire, was administered.

## Data analysis

Data were presented using frequency tables and analysed using STATA^®^ 14 (College Station, TX) statistical analytical tool. Pearson’s chi-square test was employed to assess the existence of a statistically significant association between the explanatory and dependent variables. Two logistic regression models (crude odds ratio [OR] and adjusted odds ratio [aOR]) were used to examine the extent of association between the predictor variables and family functionality. The dependent variable was binary-coded as ‘1’ for a dysfunctional family rating (moderately dysfunctional and severely dysfunctional) and ‘0’ for a functional family rating. Only significant (*p* < 0.05) explanatory variables were considered in the logistic regression model.

### Ethical consideration

Ethical approval for the study was obtained from the Committee on Human Research, Publication and Ethics (CHRPE), School of Medical Sciences (KNUST-SMS), Kwame Nkrumah University of Science and Technology and Komfo Anokye Teaching Hospital, Kumasi, Ghana (Reference Number: CHRPE/AP/014/18).

## Results

### Background characteristics of study participants

A total of 374 HIV-positive patients were recruited into the study. At the IDU of the KNUST Hospital, 126 (33.7%) participants were recruited, while 248 (66.3%) participants were recruited at the Chest Clinic of KATH. Tables 1 and 2 show the background and HIV characteristics of the participants. The majority of them were female candidates (70.1%) and the majority belonged to the Christian faith (81.8%). All the participants were in a sexual relationship and were either married (77.3%) or cohabiting (22.7%). The mean age of the participants was 41.8 years (standard deviation [s.d.] ± 9.6 years), with a minimum age of 22 and maximum age of 78 years.

**TABLE 1:** Background characteristics of study participants.

Variables	*n* = 374	% range
**Mean age (years)**	41.8	22–78
18–30	28	7.5
31–40	153	40.9
41–50	119	31.8
51–60	59	15.8
> 60	15	4.0
**Sex**		
Male	112	29.9
Female	262	70.1
**Level of education**		
Basic	242	64.7
Secondary	28	7.5
Tertiary	43	11.5
None	61	16.3
**Religion**		
Christianity	306	81.8
Islam	66	17.7
No religion	2	0.5
**Relationship status**		
Married	289	77.3
Cohabiting	85	22.7
**Number of children**		
0	28	7.5
1–4	232	62.0
> 4	114	30.5
**Employment status**		
Informal employment	294	78.6
Formal employment	48	12.8
Unemployed	32	8.6

**TABLE 2:** Human immunodeficiency virus-related characteristics of participants.

HIV characteristics	*n* = 374	% [range]
**Partner’s HIV status**		
Positive (concordant)	179	47.9
Negative (discordant)	195	52.1
**Status disclosure**		
Yes (disclosed)	350	93.6
No (undisclosed)	24	6.4
**Ways of disclosure**		
Alone at home	140	37.4
With a health worker	184	49.2
With a family member/friend/religious leader	9	2.4
Accidental disclosure	1	0.3
Met partner first time at ART clinic	10	2.7
On phone	6	1.6
None	24	6.4
**Family functionality**		
Functional	254	67.9
Moderately dysfunctional	57	15.3
Severely dysfunctional	63	16.8

HIV, human immunodeficiency virus; ART, Antiretroviral therapy.

Approximately, 93.6% of the participants had disclosed their HIV status to their sexual partners. The proportion of participants in serodiscordant relationship was 52.1% and the proportion of those who rated their families as functional was 67.9%.

### Factors associated with family functionality

Tables 3 and 4 show the relationship between background characteristics, HIV-related characteristics and family functionality. There was no statistically significant association between family functionality and participants’ age, number of children, level of education, relationship status, religion, employment status and partner’s HIV status (*p* > 0.05). Participant’s sex (*p* = 0.001) and disclosure of HIV status to partner (*p* = 0.015) were significantly associated with family functionality as shown in Tables 3 and 4, respectively.

**TABLE 3:** Relationship between background characteristics, human immunodeficiency virus characteristics and family functionality (*n* = 374).

Factors	Family functionality						*X*^2^	*p* value
	Severely dysfunctional		Moderately dysfunctional		Functional			
	*n*	%	*n*	%	*n*	%
**Age (years)**							9.3	0.318
18–30	8	28.6	4	14.3	16	57.1		
31–40	32	20.9	25	16.3	96	62.8		
41–50	14	11.8	17	14.3	88	73.9		
51–60	8	13.6	9	15.3	42	71.1		
> 60	1	6.7	2	13.3	12	80		
**Sex**							14.7	0.001
Male	7	6.3	15	13.4	90	80.3		
Female	56	21.4	42	16.0	164	62.6		
**Level of education**							8.6	0.199
Basic	44	18.2	39	16.1	159	65.7		
Secondary	6	9.8	7	11.5	48	78.7		
Tertiary	2	7.1	4	14.3	22	78.6		
None	11	25.6	7	16.3	25	58.1		
**Religion**							1.5	0.826
Christianity	50	16.3	47	15.3	210	68.4		
Islam	13	20.0	10	15.4	42	64.6		
No religion	0	0.0	0	0.0	2	100.0		
**Relationship status**							3.7	0.161
Married	43	14.9	44	15.2	202	69.9		
Cohabiting	20	23.5	13	15.3	52	61.2		
**Number of children**							0.3	0.991
0 (ref)	5	17.9	4	14.3	19	67.8		
1–4	39	16.8	34	14.7	159	68.5		
> 4	19	16.7	19	16.7	76	66.6		
**Employment status**							1.4	0.846
Informal employment	6	12.5	6	12.5	36	75.0		
Formal employment	52	17.7	46	15.7	196	66.6		
Unemployed	5	15.7	5	15.7	22	68.6		

**TABLE 4:** Relationship between background characteristics, human immunodeficiency virus characteristics and family functionality (*n* = 374).

Factors	Family functionality						*X*^2^	*p* value
	Severely dysfunctional		Moderately dysfunctional		Functional			
	*n*	%	*n*	%	*n*	%
**Partner’s HIV status**							0.8	0.683
Positive (concordant)	27	15.1	28	15.6	124	69.3		
Negative (discordant)	36	18.5	29	14.9	130	66.6		
**Status disclosure**							8.3	0.015
Yes (disclosed)	55	15.7	51	14.7	244	69.6		
No (undisclosed)	8	33.3	6	25	10	41.7		
**Ways of disclosure**							13.3	0.346
Alone at home	18	12.9	18	12.9	104	74.2		
With a health worker	34	18.1	30	16.0	123	65.9		
With a family member/friend/religious leader	3	33.3	1	11.1	5	55.6		
Accidental disclosure	0	0.0	0	0.0	1	100.0		
Met partner first time at ART clinic	1	10.0	2	20.0	7	70.0		
On phone	0	0.0	1	16.7	5	83.3		
None	7	33.3	5	23.8	9	42.9		

HIV, human immunodeficiency virus; ART, Antiretroviral therapy.

The results of the logistic regression model are presented in Table 5. In models 1 and 2, both sex and status disclosure were significantly associated with family functionality. The odds of a female HIV-positive patient rating the family as dysfunctional was less (aOR = 0.40; 95% CI = 0.24–0.69) compared with the male counterparts (Table 5). Similarly, the odds of an HIV-infected person who has not disclosed the status to his or her partner rating the family as dysfunctional was less (aOR = 0.30; 95% CI = 0.13–0.71) compared with those who have disclosed their status.

**TABLE 5:** Logistic regression model showing the relationship between gender, status disclosure and family functionality.

Predictor	Model 1 odds ratio	95% confidence interval	Model 2 odds ratio	95% confidence interval
**Sex**				
Male [*ref*]	1.00	-	1.00	-
Female	0.41†	0.24–0.69	0.40†	0.24–0.69
**Status disclosure**				
Disclosed [*ref*]	1.00	-	1.00	-
Undisclosed	0.31†	0.13 – 0.72	0.30†	0.13–0.71

ref, Reference category.

† , *p* < 0.001.

## Discussion

In this study, the proportion of HIV-infected female patients was higher than that of male patients. The Joint United Nations Programme on HIV/AIDS (UNAIDS) reports that 51% – 58% of people living with AIDS worldwide are women. In Western and Central Africa, they account for approximately 60%. Three in four new infections are amongst girls and Ghana is counted amongst the countries in sub-Saharan Africa with the highest female HIV prevalence.^12^ Gender inequity, higher biological susceptibility and intimate partner violence have been cited as some of the factors contributing to higher HIV prevalence amongst African women than men.^13^ The large surface area of the vaginal mucous membrane and suppression of their immune system during the secretory phase of their menstrual cycle increase women’s risk of infection with HIV and other Sexually Transmitted Infections.^14^ In men, circumcision provides some level of protection against acquiring HIV infection.^13^

Generally, women have a better health-seeking behaviour than men; they tend to report earlier to health facilities for the care of their illnesses than men. Often, there is a delay in diagnosis in men as compared to women.^15,16^ This study recorded 195 (52.1%) HIV-positive patients in discordant relationships. This is similar to WHO’s estimation that half of HIV-positive patients in sexual relationships have serodiscordant partners. However, a study conducted gave discordance figures between 12% and 23%, which varied across Africa.^17^ The results of this study also support previous studies which showed that for couples who are serodiscordant, there is a higher preponderance of women who are HIV-positive.^4,5^

This study assessed family instability in HIV-discordant couples using the Family APGAR tool. This is novel because it has not yet been reported in the literature. Approximately 17% of respondents in this study rated their families as severely dysfunctional, with women accounting for 57% of them. This difference was found to be statistically significant (OR 2.4; CI 1.44–4.45). There was also a statistically significant relationship between family functionality and HIV status disclosure. Female gender and partner status disclosure are therefore strong predictors of family functionality in this cohort. There was, however, no statistically significant relationship established between family functionality and HIV discordance.

Study participants who rated their families as functional were 254 in number, that is, 67.9% of all HIV-positive patients recruited in the study. Another study in Ibadan, Nigeria, amongst adolescents with risky behaviour recorded 84.5% functional family scores.^18^ The high scores recorded for family function may be evident of the fact that Ghanaians and Africans generally have been noted to have strong family ties and interdependent social support systems.

One limitation of this study is that it was cross-sectional in nature. It would be interesting to explore any change in participants’ assessment of family function over a period of time. The strength of the study is that it compared functionality in concordant couples with that in discordant couples. These data were not previously available; hence, this study provides a useful reference for future studies.

## Conclusion

This study has established that the strongest predictors of family functionality amongst HIV couples are gender and partner status disclosure. Although there was no statistically significant association with serodiscordance, report of disruption of family relationships, being upset of family members and weakened family support make it imperative that a standardised tool for assessing family function should be employed. This will equip counsellors and HIV caregivers to institute effective measures to address these issues and enhance patient survival.
